# Influence of Processing Pipeline on Cortical Thickness Measurement

**DOI:** 10.1093/cercor/bhaa097

**Published:** 2020-05-07

**Authors:** Shahrzad Kharabian Masouleh, Simon B Eickhoff, Yashar Zeighami, Lindsay B Lewis, Robert Dahnke, Christian Gaser, Francois Chouinard-Decorte, Claude Lepage, Lianne H Scholtens, Felix Hoffstaedter, David C Glahn, John Blangero, Alan C Evans, Sarah Genon, Sofie L Valk

**Affiliations:** 1 Research Centre Jülich, Institute of Neuroscience and Medicine (INM-7: Brain and Behavior), 52425, Jülich, Germany; 2 Institute of Systems Neuroscience, Medical Faculty, Heinrich Heine University Düsseldorf, 40225, Düsseldorf, Germany; 3 Montreal Neurological Institute, McGill University, Quebec, H3A 2B4, Canada; 4 Center of Functionally Integrative Neuroscience, Department of Clinical Medicine, Aarhus University, 8000 Aarhus, Denmark; 5 Department of Psychiatry and Department of Neurology, Jena University Hospital, 07747 Jena, Germany; 6 Connectome Lab, CTG, CNCR, VU Amsterdam, 1081 Amsterdam, Netherlands; 7 Department of Psychiatry, Yale School of Medicine, New Haven, CT 06511, USA; 8 South Texas Diabetes and Obesity Institute and Department of Human Genetics, University of Texas Rio Grande Valley School of Medicine, Brownsville, TX 78520, USA

**Keywords:** in-vivo cortical thickness, software comparison, reliability, replicability, interindividual variability

## Abstract

In recent years, replicability of neuroscientific findings, specifically those concerning correlates of morphological properties of gray matter (GM), have been subject of major scrutiny. Use of different processing pipelines and differences in their estimates of the macroscale GM may play an important role in this context. To address this issue, here, we investigated the cortical thickness estimates of three widely used pipelines. Based on analyses in two independent large-scale cohorts, we report high levels of within-pipeline reliability of the absolute cortical thickness-estimates and comparable spatial patterns of cortical thickness-estimates across all pipelines. Within each individual, absolute regional thickness differed between pipelines, indicating that in-vivo thickness measurements are only a proxy of actual thickness of the cortex, which shall only be compared within the same software package and thickness estimation technique. However, at group level, cortical thickness-estimates correlated strongly between pipelines, in most brain regions. The smallest between-pipeline correlations were observed in para-limbic areas and insula. These regions also demonstrated the highest interindividual variability and the lowest reliability of cortical thickness-estimates within each pipeline, suggesting that structural variations within these regions should be interpreted with caution.

## Introduction

Gray matter (GM) contains the most neuronal cell bodies in the brain, and its structure changes considerably in the course of development, aging, and in disorders. These variations can be approximated in-vivo using structural MRI by measuring variations in macroscopic properties of the cortex. Over the last decade, many studies have utilized imaging-derived macroscopic GM properties to assess neurobiological changes in the cortical structure as a function of development, aging, and pathology, as well as interindividual behavioral variations (Kanai and Rees 2011; Fjell et al. 2014, 2015; Walhovd et al. 2017).

A frequently used macroscopic feature of the cortical structure is its thickness, which characterizes the distance between the gray–white interface (inner boundary) and the pial interface (outer boundary). Despite its seemingly straightforward definition and interpretation, calculation of cortical thickness from magnetic resonance (MR) images of the highly folded human cortex is nontrivial, relying on precise identification of the boundaries between tissue types, as well as the metric used to quantify the thickness of the cortex that lies between these identified boundaries (MacDonald et al. 2000; Lerch 2001; Han et al. 2004; Lerch and Evans 2005; Das et al. 2009). In this respect, utility of manual assessments are limited, not only because they are labor intensive, but also due to the difficulty of accurate manual identification of tissue boundaries, for example, due to blurring of the gray–white boundary, as well as within highly folded areas. Therefore, it is inevitable to rely on automatic methods for characterizing cortical thickness from in-vivo MRI, in particular among large cohorts of individuals.

Currently, several software packages provide algorithms for automatic estimation of cortical thickness (such as FreeSurfer [www.surfer.nmr.mgh.harvard.edu], CIVET [http://www.bic.mni.mcgill.ca/ServicesSoftware/CIVET], Brainvoyager [https://www.brainvoyager.com], BrainVisa [http://brainvisa.info], BrainSuite [http://brainsuite.org], CAT [www.neuro.uni-jena.de/cat/], and ANTS [http://stnava.github.io/ANTs/]). These routines can be broadly divided into surface-based and volume-based approaches and are commonly validated by focusing on assessment and comparison of cortical thickness of a few selected cortical areas, with manual measurements from histological sections (Fischl and Dale 2000; Cardinale et al. 2014) from a limited number of individuals. Though these validation studies report encouraging findings about correlation of cortical thickness estimated from automatic and manual segmentations of brain structure, they also demonstrate that the thickness estimate from in-vivo MRI is only a proxy of the histological measurement of the thickness of the cortex, in particular within regions with blurred boundaries between gray and white matter.

Despite importance of such validation studies, the limited number of participants, as well as the focus on specific regions of interest (but see Wagstyl et al. (2018) for a whole cortex solution using BigBrain) and pipelines, make interpretation and generalizability of these comparisons challenging in the era of “Big Data Neuroscience.” Over the last decade, the number of participants used in structural imaging studies has increased considerably, from 10s to 1000s of individuals (Van Essen et al. 2013; Miller et al. 2016; Bearden and Thompson 2017; Smith and Nichols 2018). Such investigations often relate small submillimeter variations of the automatically determined cortical thickness to behavioral or clinical outcomes (Miller et al. 2016). These large-scale in-vivo assessments play a central role in shaping our understanding of brain variability and are thus increasing in frequency. Therefore, in the absence of an automatic pipeline, providing in-vivo ground truth of macroscale neuroanatomical variation, it is crucial to ensure reliability of the conclusions drawn from these studies through assessment of within-tool robustness and between-tool reproducibility of the estimated thickness, which can further be useful in interpreting measured macroanatomical variability as well as its modulating factors.

A handful of recent studies have attempted to perform such between-tool replicability assessment of cortical thickness estimates, within samples of healthy individuals or in patients (Clarkson et al. 2011; Gronenschild et al. 2012; Dahnke et al. 2013; Tustison et al. 2014; Li et al. 2015; Martínez et al. 2015; Dickie et al. 2017; Lewis et al. 2017; Righart et al. 2017; Seiger et al. 2018). While some studies have demonstrated high levels of within-pipeline test–retest reproducibility (Dahnke et al. 2013; Lewis et al. 2017), results of comparison of cortical thickness estimations across different versions of the same package have been less straightforward (Gronenschild et al. 2012; Dickie et al. 2017). Also, though global thickness estimates have been shown to correlate highly across different software packages (Righart et al. 2017; Seiger et al. 2018), spatial distributions of cortical thickness estimates have demonstrated marked regional between-tool differences (Clarkson et al. 2011; Li et al. 2015; Martínez et al. 2015; Dickie et al. 2017). Moreover, an investigation of brain–behavior relationship, using thickness estimates from three different pipelines (cortical pattern matching, CIVET, and BrainSuite) demonstrated considerable variations in the spatial pattern of associations between cognitive scores and cortical thickness measurements across tools, as well as irreproducible associations within tools (Martínez et al. 2015). These ambiguous reports raise concerns about the reproducibility of cortical thickness estimates and the biological validity of the conclusions that are derived from its interindividual variability.

However, calling the available cortical thickness estimation pipelines unreliable and questioning the replicability of all the literature studying variability of cortical thickness using these tools might be premature. In particular, comparing cortical thickness processing pipelines at different software development stages (i.e., beta version as opposed to stable releases; Righart et al. 2017; Seiger et al. 2018) can partly explain the differences in the derived conclusions. Furthermore, proper quality control (QC) of the outcomes as well as characteristics of the samples under study may influence the performance of the different pipelines.

In this work, we aimed to comprehensively study and compare cortical thickness estimations from three software packages with large user-based communities that generate cortical thickness estimates on the surface (FreeSurfer, CIVET, and CAT) using two large single-site datasets of healthy individuals, with different age ranges. In doing so, our approach considers potential biases due to scanning sites, population differences, and confounds due to age-related changes in macroscale GM anatomy. In each step of analysis, for example, in the group-wise registration step, we adhere to the pipelines’ originally implemented (or suggested) routine.

After visual inspection of each pipeline’s output, the quality-controlled thickness metrics were used to assess the following three main goals:

- “Within-pipeline” regional thickness variability across all participants, within each cohort.
- “Between-pipelines” thickness variability across all participants, globally and regionally, to identify regions in which cortical thickness estimations differed significantly between pipelines. Commonality of the pipelines in capturing similar “interindividual” variabilities was also assessed by means of correlations, within each sample.
- Finally, for each pipeline “test–retest reliability” of the cortical thickness estimations was assessed within a subgroup of 143 individuals.

In doing so, we aimed to provide detailed insight on global and regional robustness of cortical thickness estimations of the commonly used pipelines, generating a basic understanding about factors that may influence replicability of studies that characterize structural variations and highlighting regions in which interindividual variability in cortical thickness should be interpreted with caution.

## Methods

### Participants and Acquisition Parameters

Two large single-site datasets with high quality T_1_-weighted MRIs of healthy adults were used to assess the between-individual variability and between-pipeline replicability of cortical thickness estimation. The first dataset is the publicly available data from the Human Connectome Project (HCP; http://www.humanconnectome.org/), consisting of young healthy adults. HCP comprises data from 1113 individuals (656 females), with mean age of 28.8 years (SD = 3.7, range = 22–37). The full set of inclusion and exclusion criteria are described elsewhere (Glasser et al. 2013; Van Essen et al. 2013). Two high-resolution (isotropic 700 μm) 3D MPRAGE T_1_-weighted images were acquired using the HCP’s custom 3 T Siemens Skyra scanner with the following parameters: TE/TR/TI = 2.14/2400/1000 ms, field of view (FOV) = 224 mm, flip angle = 8°, bandwidth (BW) = 210 Hz per pixel. Two T_2_-weighted images were also acquired with identical geometry (TE/TR = 565/3200 ms, variable flip angle, and BW = 744 Hz per pixel). Full description of MRI protocols of the HCP is previously described in Glasser et al. (2013). Images underwent gradient nonlinearity correction (using acquired B1 bias field) and the two scans of each modality were coregistered and averaged. This average image is downloaded from https://wiki.humanconnectome.org/display/PublicData/.

The second dataset consisted of 902 individuals (546 females), with mean age of 43.2 years (SD = 14.8; range = 18–85) from randomly selected families of Mexican–American descent who live in San Antonio, TX, USA, enrolled in the Genetics of Brain Structure and Function Study (GOBS) before 31 December 2012 (McKay et al. 2014). The GOBS study is a collaborative effort between Texas Biomedical Research Institute, University of Texas Health Science Center at San Antonio (UTHSCSA) and Yale University School of Medicine. Exclusion criteria were MRI contraindications, history of neurological illness, stroke or other major neurological events. All MR images were acquired at the UTHSCSA Research Imaging Center on a Siemens 3 T Trio scanner (Siemens). High-resolution (isotropic 800 μm) 3D Turbo-flash T_1_-weighted images were acquired with the following parameters: TE/TR/TI = 3.04/2100/785 ms, FOV = 200 mm, flip angle = 13°, and BW = 200 Hz per pixel. Seven images were acquired consecutively using this protocol for each subject. Native T_1_-weighted MRI scans were coregistered and were further corrected for nonuniformity artifacts with the N3 algorithm (Sled et al. 1998) and averaged, for each participant over the seven scans, to increase signal-to-noise ratio (SNR) and reduce motion artifacts (Kochunov et al. 2006).

### Participants for Assessment of Within-Tool Reliability of Cortical Thickness

One hundred and forty-three participants of the GOBS cohort (100 females; mean age: 49.5 years; SD = 12.1; range = 26–81) with seven good quality scans (as defined subjectively by FCD) were selected for further analysis, to assess within-subject replicability of cortical thickness estimation for each pipeline. To increase SNR of the test–retest scans, the seven scans were divided into two groups: the “odd” and “even” acquisitions. Following estimation and correction of bias fields using the N3 algorithm (Sled et al. 1998), odd acquisitions (first, third, fifth, and seventh acquired T_1_-weighted MRIs) were coregistered (Kochunov et al. 2006). Separately, the same procedure was performed for even acquisitions (i.e., second, fourth, and sixth acquired scans). This procedure resulted in a pair of T_1_-weighted MRIs for each of the 143 participants. The pairs of scans were generated by averaging different numbers of images (four odd scans vs. three even scans), to enhance the difference in SNR between them. Comparing the cortical thickness estimations within each pair hence examines robustness of the tools to slightly different levels of noise.

### Processing Pipelines for Cortical Thickness Estimation

#### FreeSurfer Version 6.0

FreeSurfer analysis of the T_1_-weighted images for both datasets was performed using the default recon-all options (version [v] 6.0; www.surfer.nmr.mgh.harvard.edu). Briefly, the raw T_1_-weighted images are affine-registered to a 1 mm template (MNI305) and after normalization, removal of intensity bias-field and skull-stripping, white-matter voxels are identified based on intensity and neighbor constraints. The two hemispheres are then separated. By tiling the boundary of white-matter mass, an initial white surface is created, which is further refined following intensity gradients of the white and GM to generate the final gray–white surface. This surface is then expanded to follow the intensity gradient of GM and cerebrospinal fluid (CSF), fitting the pial surface.

Cortical thickness at each vertex is computed as the average of two distances, that is, from each vertex in the gray–white surface to the nearest point in pial surface and from the corresponding vertex in the pial surface to the nearest point in gray–white surface (Fischl and Dale 2000; Fischl 2012).

For the HCP data, in addition to the bias-corrected T_1_-weighted images, cortical thickness estimates derived using FreeSurfer v5.3-HCP pipeline (https://github.com/Washington-University/HCPpipelines/blob/master/FreeSurfer/FreeSurferPipeline-v5.3.0-HCP.sh) are provided through the Amazon Web Services for download. The outcome of this pipeline (Glasser et al. 2013), which is optimized for the HCP data and further incorporates T_2_-weighted images into the FreeSurfer analysis pipeline, is frequently used to report cortical thickness variations within the HCP sample. In this study, we use this opportunity to compare outcome of this customized pipeline with the thickness estimates of the FreeSurfer v6.0 default pipeline, as well as CIVET v2.1.1 and CAT v12.5, on the HCP cohort. However, due to fundamental differences between this pipeline (inclusion of additional imaging modality, as well as other modifications tailored for the HCP data), as compared with default pipeline, results of comparisons of its outcomes are only presented in the Supplementary Material and detailed discussions about the factors influencing these outcomes are considered beyond the scope of this manuscript.

#### CIVET v2.1.1

Surface-extraction and cortical thickness estimation using CIVET were performed using v2.1.1 (http://www.bic.mni.mcgill.ca/ServicesSoftware/CIVET). Briefly, T_1_-weighted MRIs are first transformed to stereotaxic MNI152 space, at 0.5 mm processing voxel resolution (Collins et al. 1994) and nonuniformity artifacts are corrected with the N3 algorithm (Sled et al. 1998) using the recommended N3 spline distance of 125 mm for 3 T T_1_-weighted scans. The bias-corrected volumes are then masked in the stereotaxic space (Smith 2002) and segmented into tissue classes (CSF, cortical GM, subcortical GM, and WM) (Zijdenbos et al. 1998), from which the partial volume estimations are derived (Tohka et al. 2004). For the purpose of the white surface extraction, the ventricles and subcortical gray tissue classes are masked as white matter, and hyperintense T_1_-weighted voxels (representing blood vessels) are masked out. The hemispheres are separated and, for each hemisphere, an initial white matter surface of genus zero is obtained using a marching-cubes algorithm, which enforces the spherical topology of the cortical mantle.

This white surface, resampled at 40 962 vertices, is then fitted to the position of the maximum local T_1_-weighted intensity gradient at the gray–white boundary. The adjusted white surface is expanded to the classified border of GM and CSF to create the pial surface (called “gray surface” in CIVET) (Kim et al. 2005).

Cortical thickness is then measured as the distance between the “white” and “gray” cortical surfaces, in the native space framework of the original MR images, using the same double average approach as previously described for FreeSurfer.

#### CAT v12.5

Unlike CIVET and FreeSurfer pipelines, estimation of cortical thickness using CAT (v2.5; www.neuro.uni-jena.de/cat/) is assessed without extensive reconstruction of the surfaces. After initial preprocessing of the T_1_-weighted images including denoising (spatial-adaptive Non-Local Means), spatial registration, bias-correction and skull-striping, the images are segmented by an adaptive maximum a posteriori approach (Rajapakse et al. 1997) with partial volume model (Tohka et al. 2004). The two hemispheres are then separated at this step and the cerebellum and hindbrain are removed, whereas the ventricles, subcortical regions and (detected) white matter hyperintensities are filled. Within each hemisphere, for every voxel in the cortical GM, the closest (without passing a different boundary) voxel on the white matter boundary is estimated. The accurate location of the final white matter boundary is then defined using the intensity gradients along the normalized vector between each GM voxel and each intermediate boundary voxel.

To avoid explicit reconstruction of the outer boundary, thickness of GM is defined directly using the distance information from the final white matter boundary, using a projection-based thickness (PBT) estimation approach. For every GM voxel, the distance to the nearest voxel in the final white matter-boundary is calculated and successors of this voxel are defined. Successor of a voxel (v1) is defined as its neighboring voxel (v2) in GM, whose distance to the white matter boundary is around one voxel &gt;v1. Accordingly, voxels with no successors are considered as local maximum and are assumed to be located at the CSF boundary; their distance from the nearest voxel in the white matter boundary results in the thickness of the cortex at that voxel. This thickness value is further corrected for GM voxels with &gt;50% CSF contribution. See Dahnke et al. (2013) for more details.

### QC of the Outcomes

Before the analyses, results of each pipeline were visually inspected to ensure their adequate quality. In particular, the extracted pial and gray–white surfaces of FreeSurfer were overlaid on each individual’s T_1_-weighted scan (in freeview; https://surfer.nmr.mgh.harvard.edu/fswiki/FreeviewGuide). For consistency with the other two pipelines, we did not perform any manual corrections on the extracted surfaces, and thus participants with gross artifacts in surface extraction (e.g., due to susceptibility artifacts in the original image, low signal, excessive movement or existence of a neurological abnormality) were discarded from further analysis.

Quality assessment for the CIVET pipeline was done in a similar manner, by overlaying each participant’s derived white and gray surfaces on its T_1_-weighted scan (in Display; http://www.bic.mni.mcgill.ca/software/Display/Display.html). Again, participants with gross artifacts in surface extraction were discarded from further analysis.

As the CAT pipeline by default does not generate the gray–pial and gray–white surfaces, quality of the outcomes was assured by visually inspecting the following three outputs per individual: (1) normalized GM segments; (2) the location of individual’s central surface, which roughly determines the 50% distance between the final white matter boundary and the boundary between GM and CSF; and (3) the spatial distribution of the *z*-scored cortical thickness for each participant. Participants with gross error in the first two criteria or spatially implausible cortical thickness distribution were then discarded from further analysis.

For the 143 individuals from the test–retest reliability analysis, visual QC was performed on outcomes of all three pipelines, for both test and retest scans. Participants with adequate quality of outcomes for all pipelines and in both scans were then retained for further analysis.

### Assessment of Regional Cortical Thickness

To perform further group level analysis, cortical thickness was regionally collapsed using the parcellation scheme of Schaefer et al. (2018). This parcellation scheme is based on the combination of a local gradient approach and a global similarity approach using gradient-weighted Markov Random models, and in the context of this study, it is used to improve SNR and interpretability of the subsequent between-software comparison.

For each individual, thickness estimations from FreeSurfer were registered to the fsaverage surface (fsaverage—163 842 vertices per hemisphere), upon which the parcels are represented. In particular, FreeSurfer uses folding pattern quantification to drive a nonlinear surface-based intersubject registration procedure that aligns the cortical folding patterns of each subject to a standard surface (fsaverage) space (Fischl et al. 1999). The registration is performed in spherical space. First, the subject’s white surface is “inflated” to the shape of a sphere, and the geometry quantification of the white surface is transferred to the sphere. The location of the vertices on the sphere is then adjusted to minimize the overall cost to establish the correspondence. For each target vertex (on the fsaverage surface), the closest source vertex in the individual’s spherical surface is found. The value of the quantity to be mapped (e.g., thickness) is then assigned from the individual’s vertex to the target vertex, thus assigning each target vertex a value (Greve and Fischl 2018).

Individuals’ thickness estimates from the CAT pipeline were also registered to fsaverage space, via a surface registration approach using anatomic features of cortical depth and curvature information (Yotter et al. 2011).

The standard surface within the CIVET pipeline is the MNI-ICBM152 symmetric surface (with 40 962 vertices per hemisphere). Surface registration is performed to resample each individual’s cortical measurements to this template surface (akin to fsaverage in FreeSurfer), enabling vertex-wise group comparisons and application of surface parcellations for regional analyses. To assess cortical thickness estimation of the CIVET pipeline from the same regions as the other pipelines, registrations between the MNI-ICBM152 template surface and fsaverage were assessed, allowing projection of Schaefer’s parcellation on the MNI-ICBM152 template (Lewis et al. 2019).

Here, we demonstrate the regional analysis using 400 parcels. For each participant, regional cortical thickness is assessed as a trimmed mean (10%) of thickness estimates within each parcel. Global thickness, per participant, is assessed as the mean of regional cortical thickness estimations over all parcels.

### Statistical Analysis

For each sample, the “within-pipeline” estimates of cortical thickness estimation are summarized in terms of the distribution of the global thickness, spatial distribution of regional means, and SD, across individuals. The standard-deviation maps depict regions in which each pipeline demonstrated the highest variability across participants of one cohort. Comparison of these spatial maps and global estimates provides insight about commonalities and differences between cohorts and across different pipelines.

“Between-pipeline” comparison of the cortical thickness estimations is further investigated using paired *t*-tests, within each region as well as on the global thickness estimates. Two-sided significance threshold was set on *P* < [0.05/(number of parcels × number of pairs of tools)], to identify regions with significantly different cortical thickness estimation between each pair of pipelines.

Furthermore, to investigate the degree to which each pipeline-pair depicts similar interindividual variations, Spearman’s rank correlations were assessed among tools, both globally, showing overall consistency of the cortical thickness estimations, and regionally, depicting regions with highest and lowest divergence between processing pipelines.

#### Assessment of Test–Retest Reliability of Cortical Thickness Measurements

Evaluation of reliability of cortical thickness estimations within each pipeline is also performed at both the global and regional level. For each pipeline, mean absolute percent error (MAPE %), over all test–retest participants, is measured using the following formula:

$$ \left(\mathrm{MAPE};\%\right)=\left(\frac{1}{n}\sum \frac{\left|{c}_1-{c}_2\right|}{\frac{\left({c}_1+{c}_2\right)}{2}}\right)\times 100 $$$$

where ${c}_1$ and ${c}_2$are the cortical thickness estimations (global or parcel-wise cortical thickness) of the two average scans for each participant, and *n* denotes the number of participants. Given that the two average scans belong to the same participant, their cortical thickness estimates should, in theory, be identical. Therefore, the observed variations will be considered as measurement errors.

## Results

### General Qualitative Remarks from Visual QC

About 4602 surface reconstructions, 2301 volumetric segmentations, and spatial cortical thickness-distribution maps were visually inspected to ensure the quality of the processing outcomes. Both datasets used in this study had high quality T_1_-weighted MRIs, with very few images being affected by motion artifacts. Within the HCP sample, 75 participants were discarded due to anatomical abnormalities, severe movement artifacts or other technical problems (see https://wiki.humanconnectome.org/pages/viewpage.action?pageId=88901591, issue codes A and B, for detailed explanation and examples of such issues). Ten additional participants were excluded following the visual QC of the FreeSurfer outcomes, whereas 21 participants were excluded following the visual QC of CIVET results. An additional 24 participants were excluded following the inspection of spatial distribution of cortical thickness estimates from the CAT-pipeline, resulting in a sample of 981 participants for further comparisons.

From the total of 902 GOBS participants, 30 individuals with substantial surface extraction errors were discarded in the FreeSurfer pipeline. Additionally, 10 were discarded in the CIVET pipeline and 30 participants were discarded after QC of CAT results, due to abnormal regional distribution in cortical thickness, leaving 832 participants from the GOBS cohort for further analysis.

These exclusions were caused by gross errors in the processing pipelines. Modest imperfections, such as small issues with brain extraction, specifically in the postcentral gyrus within the FreeSurfer pipeline, and infrequent defects in white surface extraction resulting in generation of bridges across a sulcus in the CIVET pipeline, were ignored (see Supplementary Fig. 1*A*(ii–iv)). Also, both the FreeSurfer and CIVET pipelines occasionally showed difficulties in placing the inner surface (gray/white boundary) accurately near the insular cortex (e.g., see Supplementary Fig. 1*A*(i) and *B*(i)). Table 1 summarizes demographic characteristics of the remaining participants.

**Table 1:** Demographic characteristics of samples under study

	*n* (% female)	Age (mean ± SD)
HCP sample	981 (55%)	28.8 ± 3.6 [22–37]
GOBS sample	832 (60%)	402.7 ± 14.6 [18–85]
Test–retest sample	115 (70%)	49.3 ± 11.44 [27–77]

#### Distribution of Regional and Global Mean Cortical Thickness and Its Variability Across Participants

**Figure 1:**
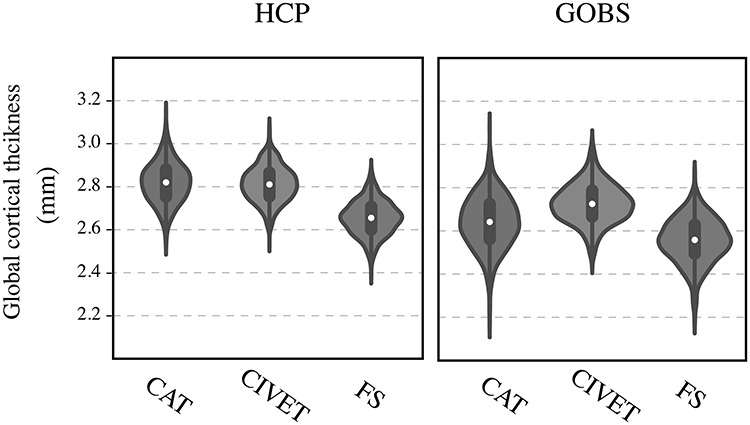
Distribution of global cortical thickness across samples and pipelines. FS: FreeSurfer.

In both datasets, CAT and CIVET resulted in higher global cortical thickness estimations, compared with FreeSurfer (Table 2 and Fig. 1). However, the difference between global cortical thickness estimations using CAT and FreeSurfer was less pronounced in GOBS cohort compared with the participants of the HCP sample.

**Table 2:** Distribution of global cortical thickness, across all participants of each sample (mean ± SD)

	HCP	GOBS
Fs 6.0	2.66 ± 0.077 [2.4–2.90]	2.56 ± 0.1 [2.2–2.87]
CAT	2.82 ± 0.09 [2.53–3.17]	2.64 ± 0.12 [2.18–3.09]
CIVET	2.81 ± 0.08 [2.54–3.07]	2.72 ± 0.09 [2.45–3.02]

**Figure 2:**
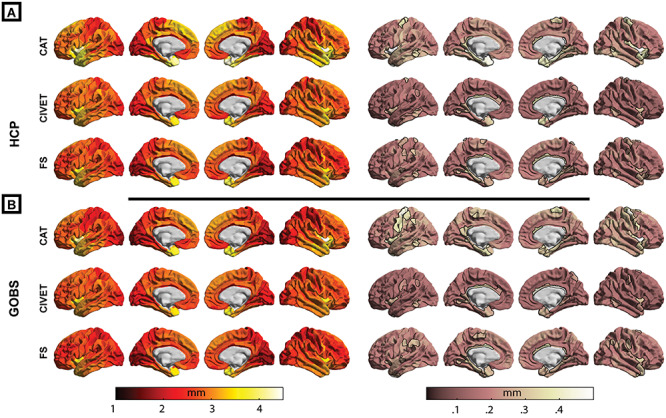
Regional mean (left columns) and SD (right columns) of cortical thickness estimates of each pipeline. For each pipeline, parcels with the highest SD (top 10%) are depicted with a black surrounding.

Spatial distributions of mean and standard deviation of regional cortical thickness across individuals, for each pipeline, are shown for HCP and GOBS samples in Figure 2. Overall, for all pipelines, cortical thickness showed the expected distribution of thinner estimated cortex in the visual and somatosensory cortices, while insular cortex, temporal poles as well as dorsal–medial prefrontal cortex showed highest cortical thickness measurements.

For comparison, Supplementary Figure 2*A* demonstrates the distribution of the width of the cortical mantle (only on the left hemisphere) as measured by von Economo and Koskinas, by means of histological examinations of postmortem brains (age: 30–40 years) (von Economo and Koskinas 1925). For detailed information about the manual segmentation of the von Economo–Koskinas atlas onto individual T_1_ scans and the subsequent construction of the digital atlas, see Scholtens et al. 2016. Accordingly, the thickest cortex in their experiments was measured in the motor and premotor regions as well as the inferior medial temporal lobe, while the thinnest cortex was measured in the postcentral gyrus and occipital lobe. Despite differences, specifically in the insular cortex and cingulate area, these results partly corroborate the distribution we observed in the MR-derived thickness estimates using all pipelines. The similarity of the spatial distributions between MR-derived cortical thickness and the histological assessments are quantified in Supplementary Figure 2*B*, for the HCP sample. Here, labels of the von Economo–Koskinas atlas are identified on the fsaverage surface. Regional cortical thickness of each pipeline is then identified using similar procedure, as described for the Schaefer’s parcels. In line with a previous publication (Scholtens et al. 2015), we observed moderate correlations (ranging between 0.57 and 0.66) between in-vivo thickness estimates of all pipelines and the histological measurements.

Considering the variability of the regional cortical thickness across participants, CIVET and CAT showed the lowest and highest regional SD, respectively.

In all tested pipelines, we observed highest SD in cortical thickness estimates in insular cortex, medial temporal pole, parahippocampal gyri, cingulate gyri, and temporo-parietal junction across participants and cohorts. Furthermore, in both samples, CAT showed high SD in regions around the central sulcus (i.e., pre- and postcentral gyrus, specifically in the left hemisphere; see Fig. 2). For each cohort, tables of mean and SD of cortical thickness within each parcel are shared (https://github.com/shahrzadkh/CT_replicability_reliability), for all three tested pipelines.

### Between-Pipelines Comparison of Cortical Thickness Estimations

#### Comparison of the Actual Thickness Estimates

**Figure 3:**
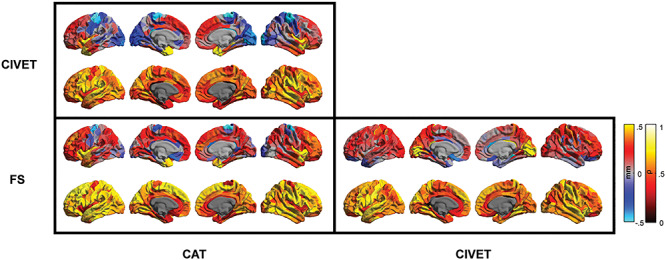
Regional between-pipeline comparison of cortical thickness estimates within the HCP sample. Top rows: paired-difference map of cortical thickness estimates; red-yellow colors depict higher thickness estimate of the pipeline mentioned in column versus row and the dark-light blue depicts the opposite direction. Only regions with significant paired *t*-test comparisons are colored (*P* < 4 × 10^−5^); lower rows: between-pipeline regional Spearman’s correlation.

**Figure 4:**
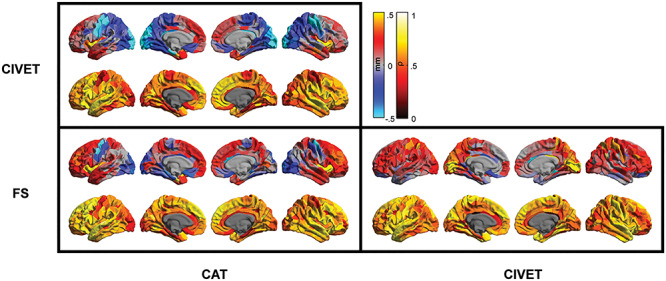
Regional between-pipeline comparison of cortical thickness estimates within the GOBS sample. Top rows: paired-difference map of cortical thickness estimates; red-yellow colors depict higher thickness estimate of the pipeline mentioned in column versus row and the dark-light blue depicts the opposite direction. Only regions with significant paired *t*-test comparisons are colored (*P* < 4 × 10^−5^); lower rows: between-pipeline regional Spearman’s correlation.

Regional paired *t*-test comparison of the cortical thickness estimates showed cortex-wide significant differences between all pipelines. Figures 3 and 4 show HCP and GOBS samples, respectively. Across both samples, CAT demonstrated consistently lower cortical thickness estimates within the pre- and postcentral gyrus as well as within the visual cortex, compared with CIVET and FreeSurfer. In contrast, insular cortex and temporal lobes were estimated systematically thicker in CAT. Relative to both FreeSurfer versions (see also Supplementary Fig. 5), CIVET estimated the cortex as thicker, specifically within the medial occipital lobe. While thickness estimations of CAT and CIVET were minimally different in the dorsal lateral frontal lobes, FreeSurfer comparably resulted in much lower thickness estimates within this region, in both samples.

**Figure 5:**
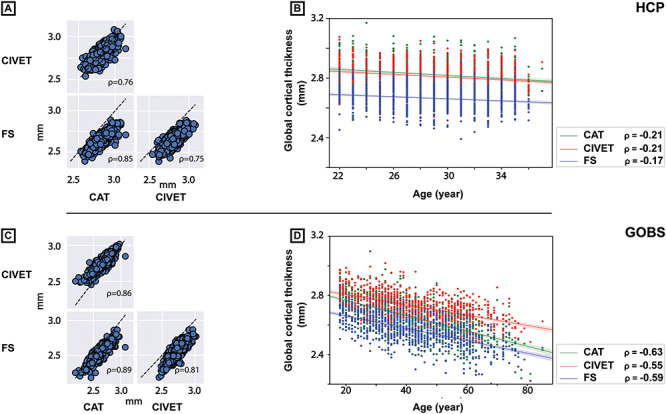
Between-pipeline comparison of global cortical thickness and its interindividual variability. Scatter plots of pair-wise comparison of global cortical estimates and their Spearman’s correlations, within two cohorts. (*A*, *C*) Dashed line depicts the identity line(*y* = *x*). (*B*, *D*) Scatter plots of association between participants’ age and global cortical thickness from each pipeline, for each cohort. Legends depict Spearman’s correlation between age and global thickness of each pipeline.

As depicted in Supplementary Figure 5, within the HCP sample, the two FreeSurfer processing pipelines also showed slight differences; thicker medial structures and thinner parahippocampal gyri and medial temporal poles were measured by FreeSurfer v5.3-HCP compared with the standard pipeline of FreeSurfer v6.0.

#### Between-Pipelines Correlation of Cortical Thickness Estimates

Despite the differences in the absolute value of thickness estimations between pipelines, correlation analysis indicated that interindividual variabilities of the estimated global cortical thickness correlated highly across all pipelines (all correlations > 0.75; Fig. 5*A,C*), in both HCP and GOBS datasets. Accordingly, global cortical thickness in all pipelines correlated similarly with age of participants, within both samples (HCP: age-correlations $\approx$ −0.2; GOBS: age-correlations $\approx$ −0.6; Fig. 5*B*,*D*), emphasizing again that the interindividual variabilities are depicted similarly in the global thickness estimates.

**Table 3:** Test–retest results MAPE

	Mean ± SD	Min	25%	50%	75%	Max
FS 6.0	2.488 ± 0.899	1.4	2.02	2.28	2.68	11.32
CAT	2.18 ± 1.1	0.945	1.51	1.85	2.51	9.11
CIVET	2.026 ± 1.09	1.07	1.54	1.77	2.14	14.9

**Figure 6:**
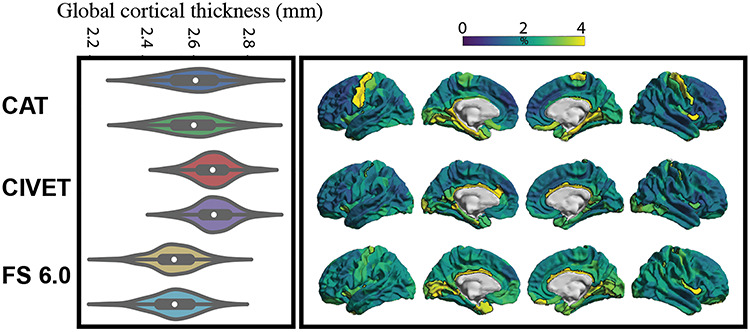
Test–retest reliability. Left: pairs of violin plots demonstrate distribution of global cortical thickness estimates from odd and even scans, over all the subjects. Right: regional distribution of MAPE for each pipeline. The top ten percent of the parcels, showing the lowest test–retest reliability, are surrounded with black line. The lighter colors in the spatial maps depict higher MAPE.

Regionally, in the HCP sample, thickness estimates of the two FreeSurfer versions correlated highly within most cortical parcels, with the exception of medial frontal lobe, insular cortex, parahippocampal gyri, and medial temporal poles (Supplementary Fig. 5). Considering in addition the CAT and CIVET pipelines, cortical thickness estimates of the medial frontal cortex, insular cortex, cingulate, precentral and parahippocampal gyri, as well as temporal poles showed lower correlations between all the pairs of tested pipelines in this cohort (Fig. 3). In contrast, cortical thickness estimates within postcentral gyrus and superior temporal lobes showed highest between-pipeline correlation in this sample. Comparable observations were made in the GOBS cohort. In particular, cortical thickness estimation of CAT showed lowest correlation with CIVET and FreeSurfer estimates within the orbitofrontal and precentral gyri, superior parietal and occipital lobes, and parahippocampal and cingulate gyri (Fig. 4). Lowest correlations between CIVET and FreeSurfer thickness estimates were found in the dorsal lateral frontal lobe, parahippocampal and cingulate gyri as well as in the parietal lobule. In line with the HCP sample, highest correlations were found in the postcentral gyrus and superior temporal lobes.

Importantly, most regions with low between-pipeline correlations, including the parahippocampal and cingulate gyri, insular cortices, precentral gyrus, and occipital lobes, also showed high within-sample standard deviation of thickness estimates in each pipeline (comparing Figs 3 and 4 with Fig. 2).

Regional associations between cortical thickness and age of participants are also demonstrated in the SupplementaryFigure 10, for both cohorts. Accordingly, these maps show a similar pattern of association across all pipelines, while differences are most visible within regions in which we have observed the lowest between-pipeline correlation of the thickness estimates, namely insular cortices, regions around the central sulcus, occipital lobes, and temporo-parietal junction.

### Test–Retest Reliability of Cortical Thickness Measurements

Out of the 143 participants for the test–retest analysis, visual inspection defined 115 individuals to have adequately good quality following processing by all three tested pipelines, and for both test–retest scans (2/5 participants excluded from FreeSurfer v6.0, 5/5 participants excluded from CIVET v2.1.1, and 6/8 individuals excluded from CAT v12.5, referring to the odd [first] and even [second] scan groups, respectively).

All three tested pipelines showed high test–retest reliability. In particular, pairwise Spearman’s correlation coefficients between the global cortical thickness estimations of both the test and retest scans (i.e., odd and even scans) were >0.96 ($\rho$_CAT_: 0.99; $\rho$_CIVET_: 0.97; $\rho$_FreeSurfer_: 0.96). Regional distribution of MAPE, demonstrating the relative measurement error, showed that for all tested pipelines, >50% of the parcels had a MAPE <3% (Table 3).

Insular cortex, medial temporal pole, parahippocampal gyri, and cingulate gyri showed reduced reliability of thickness estimations using all three tested pipelines (MAPE in these areas reach >10%). In addition, CAT showed reduced reliability within the precentral gyrus and FreeSurfer demonstrated higher MAPE, that is, reduced reliability, also in medial occipital lobe (Fig. 6).

## Discussion

In this work, we investigated reliability and replicability of cortical thickness estimations from three software packages with large user-based communities (FreeSurfer, CIVET, and CAT) using two large single-site datasets of healthy individuals. While all three pipelines, on average, demonstrated a similar pattern of thickness distributions throughout the cortex, the estimated values differed significantly between pipelines. Using pair-wise comparisons in both samples, we highlighted regions in which each pipeline tends to under- or overestimate cortical thickness relative to the two other pipelines. At the same time, interindividual variability of cortical thickness covaried strongly among tools, suggesting relatively consistent under- or overestimations across pipelines. Last, test–retest analyses demonstrated high reliability of the thickness estimations in most parts of the brain within each processing pipeline. However, regions with low between-pipeline correlation, mainly located in the parahippocampal and cingulate gyri, insular cortex, precentral gyrus, and occipital lobe, consistently demonstrated higher interindividual variability, as well as low test–retest reliability, within each pipeline. In sum, using a big-data approach to reliability and replicability of cortical thickness, we observed that while interindividual variabilities in cortical thickness estimates are comparably depicted across samples and pipelines, absolute values differ. Moreover, estimates of thickness in para-limbic and midoccipital regions vary most within, as well as across individuals and also across pipelines. These results suggest that interindividual variability of the cortical thickness within these regions is less reliably estimated and should be interpreted carefully.

### Regional Distribution of Cortical Thickness Estimations

In two large samples of healthy individuals, and across processing pipelines, regional cortical thickness estimates demonstrated a similar distribution pattern, with thinner cortex estimated in the visual and somatosensory regions and thicker cortex within insular cortex, temporal poles as well as medial prefrontal cortex and premotor areas. These observations are in line with the previous findings in the literature and histological studies, demonstrating lower thickness of the cortical mantle in the somatosensory regions as well as in the visual cortex (Rowley et al. 2015). Notably, we observed a positive correlation between MRI-derived measures of cortical thickness, using all three pipelines, and width of the cortical mantle as reported in the histological work of von Economo and Koskinas (1925). We acknowledge that the accuracy of this analysis is restricted by lack of correspondence between participants used for in-vivo and ex-vivo examinations and further inherent limitations of most ex-vivo measurements, including use of tissue samples from only a limited number individuals with unknown amounts of underestimation due to postmortem volume shrinkage and overestimation due to selected sectioning procedure (Amunts et al. 2007; Scholtens et al. 2015). Despite these limitations, our results suggest a moderate level of agreement ($P>0.57$) in the spatial distribution pattern of the cortical thickness, albeit with different absolute values, between the histological and MR-derived estimates, using all three pipelines.

At the same time, we observed that, in all samples and across pipelines, and unlike the histological assessments (see Supplementary Fig. 2), insular cortex and the temporal pole were the regions with the thickest cortex in MR-based measurements. Visual QC showed that these regions are also particularly prone to errors. For example, Supplementary Fig. 1*B* (i) depicts one such failure, where the thin white matter between insula and claustrum is missed and hence the white surface of the posterior insula is misplaced. Similarly, the thin white matter within the temporal lobe and relatively higher impact of partial volume effects between gray and white matter within these regions, may lead to misplacement of white surface inside white matter. Such occasional imperfect surface definitions can in turn result in inflated cortical thickness estimates within these regions and its higher variability across participants.

Differences between in-vivo and ex-vivo thickness estimations can also arise from inherent limitation of the T_1_-weighted MRI signal (Natu et al. 2019). For example, higher myelin content within motor and premotor areas increases the intensity of voxels in T_1_-weighted anatomical MR images, shifting the apparent gray–white boundary in these regions and thus resulting in underestimation of cortical thickness from in-vivo measurements, in all pipelines.

### Distribution of Interindividual Variability of Cortical Thickness Estimates

Our results demonstrated an important aspect of interindividual variability that is depicted in standard deviation maps by all pipelines. In both young and older adults, all pipelines showed high interindividual variation of thickness within the posterior cingulate gyri, parahippocampal gyri, temporal pole, insular cortex, and temporo-parietal junction. The pattern of spatial distribution of average cortical thickness over all participants and its variation, within each tool, was confirmed in both HCP and GOBS sample, highlighting the replicability of these findings despite the characteristic differences between the two samples.

This observation may reflect true biological interindividual variability of these regions. Alternatively, the consistently higher variability in cortical thickness estimations within these regions might demonstrate higher frequency of processing errors that can occur in these areas. Our test–retest analyses, demonstrating low reliability of cortical thickness estimations within these regions, support this later explanation. This suggests that the heightened interindividual variability in these areas is, at least partly, driven by common difficulty of all pipelines in correctly characterizing tissue boundaries in these regions. Specific structural properties, such as very thin local structures (white matter and/or CSF) or lower contrast in the MR-images are possible factors that can negatively influence the accuracy of automatic pipelines in correctly identifying the tissue boundaries of these regions. Accordingly, our results highlight that cortical thickness estimates within these regions may be prone to errors and are thus less reliable. Higher variability of estimated cortical thickness within these less reliable regions can, in turn, obscure true biological variations and result in spurious findings, for example, in studies linking brain structure to variation in nonbrain phenotypes (Loken and Gelman 2017), but also group-wise differences. As such, findings within these regions should be taken with caution.

### Between-Pipeline Comparison of Cortical Thickness Estimation

In line with previous work (Lerch and Evans 2005; Dahnke et al. 2013; Dickie et al. 2017; Lewis et al. 2018), we showed that different pipelines produce different absolute values of cortical thickness estimates, highlighting the dependency of in-vivo cortical thickness estimates on the processing software and the metric that is used to estimate the thickness of the cortex. Thus, in-vivo values of cortical thickness are only a proxy of the actual thickness of the cortex and are limited to the specific analysis pipeline (e.g., Alvarez et al. 2019). These differences complicate straightforward use of cortical thickness estimates, acquired from different pipelines, in a single study.

For example, unlike CIVET and FreeSurfer pipelines, cortical thickness in CAT v12.5 is defined using a PBT estimation approach. Calculating the thickness using the same double average approach that is implemented in FreeSurfer (Tfs), instead of the PBT, would result in lower thickness values throughout the cortex. Influence of this modification is demonstrated in Supplementary Figures 3–8. As these figures show, CAT cortical thickness using Tfs approach results in smaller values globally and in all cortical areas, across participants, bringing CAT’s thickness estimates in the range of FreeSurfer’s measurements. Yet, despite this change in the absolute value of thickness, correlations between the modified CAT-thickness estimates and CIVET and FreeSurfer outcomes remain unchanged, showing that the modification to the thickness measurement approach has not influenced the pattern of interindividual variabilities.

Importantly, we observed that the difference between pipelines is not uniformly distributed across the cortical mantle. Irrespective of the thickness estimation approach, CAT underestimated thickness around the central sulcus, compared with the other pipelines. The underestimation of the cortical thickness around the central sulcus and specifically in motor cortex, might be attributed to its proportional higher myelin content (Rowley et al. 2015), which as mentioned earlier results in higher intensity of voxels in T_1_-weighted images and influence the apparent gray/white contrast in this regions. While this effect can result in underestimation of the cortical thickness in all MR-based automatic pipelines, it is particularly problematic for CAT, which relies strongly on actual intensities of the MR images for identification of the white matter boundary (Dahnke et al. 2013). CIVET, on the other hand, estimated the cortex systematically slightly thicker than both the FreeSurfer pipelines. This is in line with recent observation, comparing surface estimates of CIVET v2.1.1 and FreeSurfer v6.0 with ground-truth measurements from BigBrain (Lewis et al. 2018). Their findings demonstrate that both pipelines extract a similar and relatively accurate white surface (especially at higher resolutions), but that CIVET commonly overestimates the gray surface placement (yielding higher thickness values), while FreeSurfer commonly underestimates the gray surface placement (yielding lower thickness values). Accordingly, findings of this study suggest that such over- and underestimations in both pipelines are also occurring in in-vivo measurements, resulting in systematically thicker thickness estimates in CIVET, compared with FreeSurfer estimates.

Comparing the two FreeSurfer pipelines (Supplementary Fig. 5), v5.3-HCP resulted in thicker medial structures and thinner parahippocampal gyri and medial temporal poles, compared with the default v6.0.

Though our results indicate tool-based differences in the cortical thickness estimates within each participant, pair-wise correlations suggest that interindividual variation that is depicted by these pipelines is comparable. In particular, cortical thickness estimates within the postcentral gyrus, the superior-temporal lobes, as well as the lateral frontal lobes showed the strongest between-pipelines correlation.

Comparability of the outcomes of neuroimaging software, in particular in the absence of in-vivo ground-truth measurements*,* plays a central role in the discussions about replicability of neuroscientific findings (Muhlert and Ridgway 2016). In this regard, previous studies showing strong discrepancies of the outcomes from widely used cortical thickness estimation pipelines are particularly alarming (Martínez et al. 2015; Dickie et al. 2017). However, our comparative examinations on two samples from two sites and with different characteristics, accompanied by thorough quality inspection, suggest that estimated thickness from different pipelines show high level of correlation globally and in most brain regions. It is thus likely that lack of reproducibility in studies looking at between-subject variations in cortical thickness, defined using either of these pipelines, in association with behavior or disease status cannot be solely attributed to the difference in applied software.

Quality assurance of the outcomes of neuroimaging software is an important, yet frequently overlooked task, particularly in studies with high numbers of participants. Accordingly, careful exclusion of participants with suboptimal processing outcomes for each tool is best achieved by interactively visualizing outcome surfaces, as opposed to quality assurance using selected screenshots of the surface overlays. This may partially explain the superior similarity between cortical thickness estimations of the three tested pipelines in this study as compared with some previous studies (Martínez et al. 2015; Dickie et al. 2017).

Yet, the spatial maps of pair-wise correlations also highlighted regions in which cortical thickness estimates of different pipelines correlated poorly. Medial orbitofrontal cortex, cingulate and parahippocampal gyri as well as occipital lobe showed the lowest correlations in all comparisons and within both samples. Furthermore, most regions that showed high standard deviation within one pipeline (e.g., regions around the central sulcus, showing high SD in CAT pipeline) also showed lower between-pipeline correlations, suggesting that the variability in those brain regions are potentially driven by noise and errors and therefore depicted differently by different pipelines.

### Test–Retest Reliability of Cortical Thickness Measurements and Influence of Noise

Test–retest reliability of neuroimaging outcomes is the stepping stone toward reliable science, and its importance is on par with between-pipeline replicability of outcomes. In this regard, our results showing near perfect (i.e., MAPE < 3%) test–retest reliability of cortical thickness estimation across most brain regions, for all tested pipelines, are very encouraging. It is important to note that to assess robustness of the cortical thickness estimations to slight differences in noise level, we generated the two test–retest scans by averaging different numbers of MR images. Similarity of the cortical thickness estimates between these two scans, despite the possible different noise levels, confirms that all the tested tools can robustly deal with small variations of noise in the input images.

Another informative aspect of the test–retest analysis is the regional distribution of MAPE. In particular while all pipelines had very high reliability (i.e., <2% variation) in the frontal and superior temporal lobes, the stability of the cortical thickness estimates is lower within the insular cortex, temporal poles, temporo-parietal junction, posterior-central and cingulate gyri, as well as in occipital lobes. Also, irrespective of thickness estimation metric, regions around the central sulci and parahippocampal gyri showed lowest reliability in CAT. As stated earlier, these regions also show the highest interindividual variability within each pipeline (see Fig. 2 and Supplementary Fig. 9) and have the lowest between-pipeline correlations. This demonstrates that the cortical thickness estimates within these regions are less reliable and thus calls for particular caution in interpreting findings within these regions.

## Future Research

In this work, we compared three surface-based thickness estimation methods with a large user base (FreeSurfer, CIVIT, and CAT). It is important to note that differences in analysis pipelines to estimate thickness are not trivial, and there is variability at various levels of analysis. For instance, differences in the algorithms used for the skull-stripping or brain extraction, initial- and final voxel-based registrations that can impact extraction of hemispheres and filling of ventricles and subcortical structures, and different bias-correction and tissue-classification algorithms, as well as the algorithmic solutions to dealing with geometric constraints, and alignment approaches all are likely to impact estimates of cortical thickness. As this work focused on studying thickness estimates as a result of complete analysis pipelines from different software, future research should further disseminate the effects of each processing step on thickness estimation outcomes. Moreover, in this work, we compared thickness estimates at return on interest-level to improve SNR and interpretability of the subsequent between-software comparison. Further studies should systematically compare parcel versus vertex-wise estimates of cortical thickness and the regional variation of surface-based alignment, to further qualify and quantify differences in thickness estimations across estimation approaches.

## Summary and Concluding Remarks

Using two large samples of younger and older healthy adults and three widely used packages for cortical thickness estimation, this study addressed two fundamental factors that can potentially compromise the reproducibility of neuroscientific findings: similarity of cortical thickness estimations between-pipelines, and test–retest reproducibility of cortical thickness estimations within-pipelines.

Our investigations demonstrated that (1) estimates of the three tested packages, on average, result in a similar spatial pattern of cortical thickness distribution, suggesting that the relative thickness estimates of the cortical areas are comparably depicted by these three pipelines; (2) actual measured thickness differs significantly between-pipelines, in most cortical areas, indicating that in-vivo thickness measurements are a proxy of actual thickness of the cortex, and actual values shall only be compared within the same software package and thickness estimation technique; (3) despite the different actual values estimated by different pipelines, in most cortical areas the interindividual variability is depicted comparably by different tools, supporting general replicability of the findings of studies, assessing interindividual variability in cortical thickness, using either of these pipelines; (4) furthermore, we observe a high level of within-tool test–retest reliability of cortical thickness in all three pipelines, demonstrating a general high level of reliability of the estimates within each pipeline; (5) yet, at the same time, we observed that in insula, cingulate gyrus, medial occipital lobe, parahippocampal gyrus, and medial temporal pole; cortical thickness is estimated less reliably. Accordingly, these results imply regional variation in reliability and replicability of cortical thickness indices, and encourage attempts seeking to reproduce findings about variation in structural properties, particularly, within these less reliable regions.

## Funding

The Deutsche Forschungsgemeinschaft (DFG, GE 2835/1-1 and EI 816/4-1), the Helmholtz Portfolio Theme “Supercomputing and Modelling for the Human Brain,” and the European Union’s Horizon 2020 Research and Innovation Programme under Grant Agreement No. 720270 (HBP SGA1) and Grant Agreement No. 785907 (HBP SGA2).

## Notes

The authors thank the Compute Canada (https://www.computecanada.ca) and the Jülich Supercomputing Centre (https://www.fz-juelich.de/ias/jsc/) for the usage of the computing facilities in the development of this work. Data were provided [in part] by the HCP, WU-Minn Consortium (Principal Investigators: David Van Essen and Kamil Ugurbil; 1U54MH091657) funded by the 16 NIH Institutes and Centers that support the NIH Blueprint for Neuroscience Research; and by the McDonnell Center for Systems Neuroscience at Washington University. *Conflict of Interest*: None declared.

## Supplementary Material

Updated_Supplementary_Figures_bhaa097
