# Supplementary material for: Influence of Processing Pipeline on Cortical Thickness Measurement
Source: Cereb Cortex. 2020 May 7;30(9):5014–27. doi: 10.1093/cercor/bhaa097 (PMC7391418; doi:10.1093/cercor/bhaa097)
Supplement: Updated_Supplementary_Figures_bhaa097 [file updated_supplementary_figures_bhaa097.pdf]

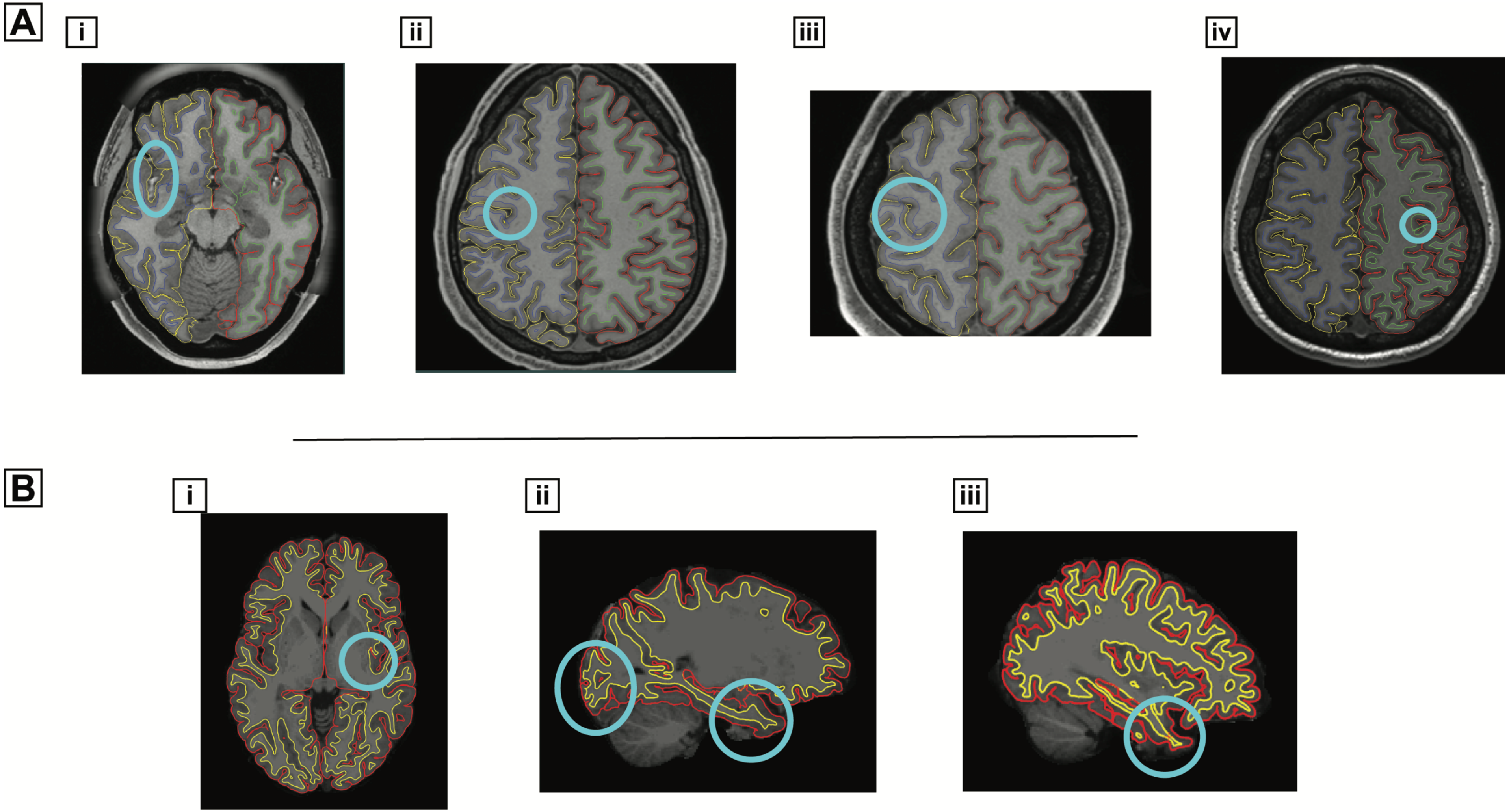

Supplementary Figure I Several examples of errors in the CIVET (A) and FreeSurfer (B) pipelines.

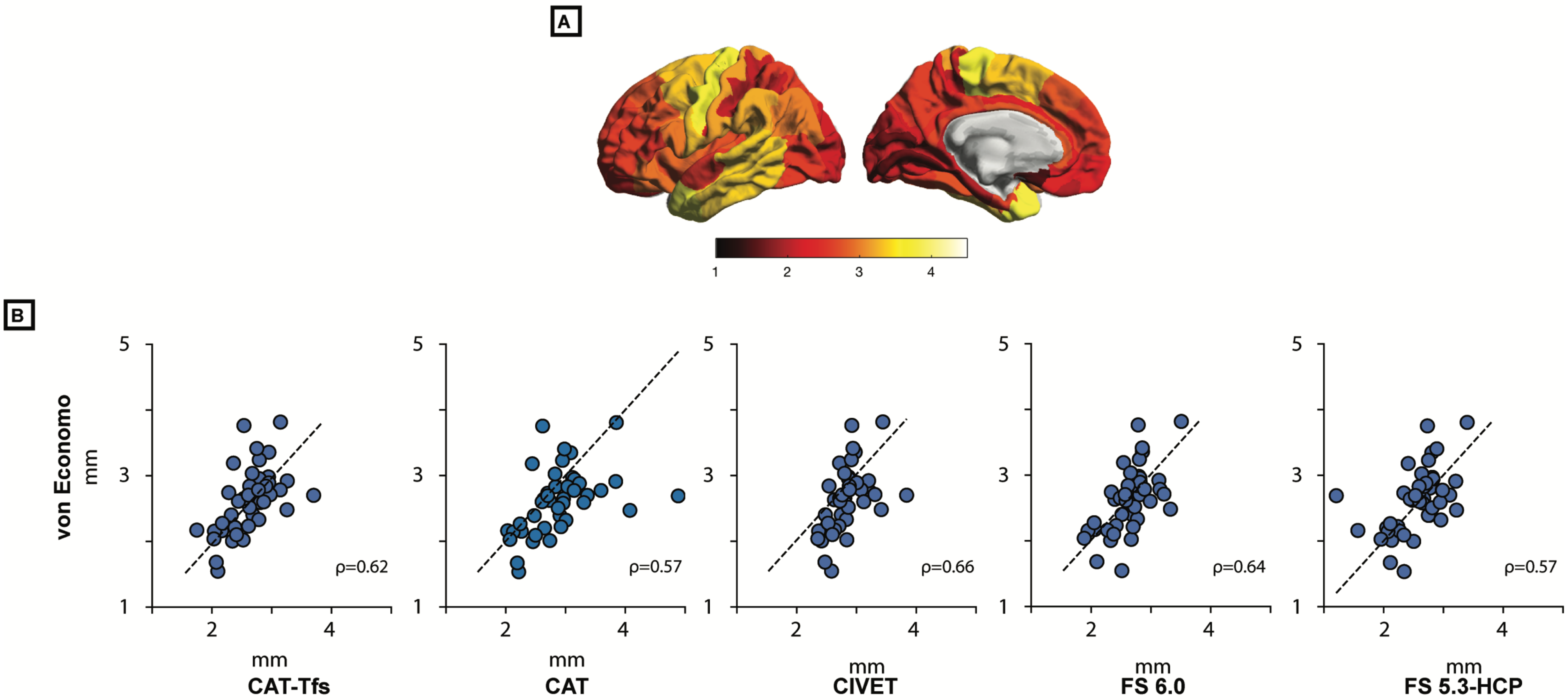

Supplementary Figure II Comparison with the manual measurements. Von Economo and Koskinas' reported cortical width values, within the boundaries of the manually segmented von Economo (1925) regions on fsaverage space (A); Scatter plots and Spearman's correlation between von Economo and Koskinas' reported cortical width values in all regions and thickness estimates from each automatic pipeline, within HCP cohort (B; dashed lines depicts the identity line(y=x)). CAT-Tfs: modified CAT thickness estimates-Tfs; FS: FreeSurfer.
For detailed information about the regions of the von Economo-Koskinas atlas, see http://www.dutchconnectomelab.nl/economo/.

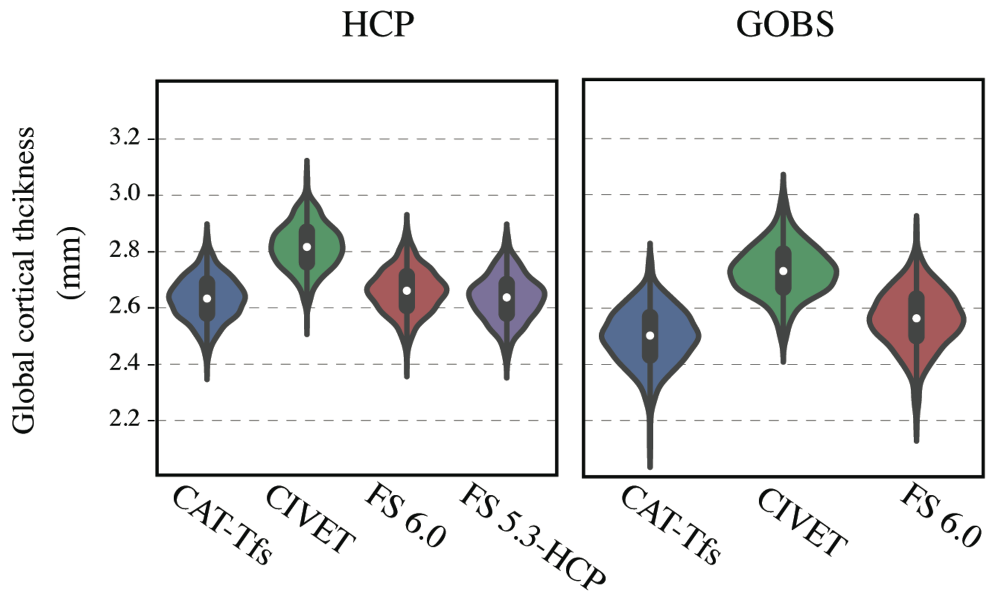

Supplementary Figure III Distribution of global cortical thickness across samples and pipeline. CAT-Tfs: modified CAT thickness estimates-Tfs; FS: FreeSurfer.

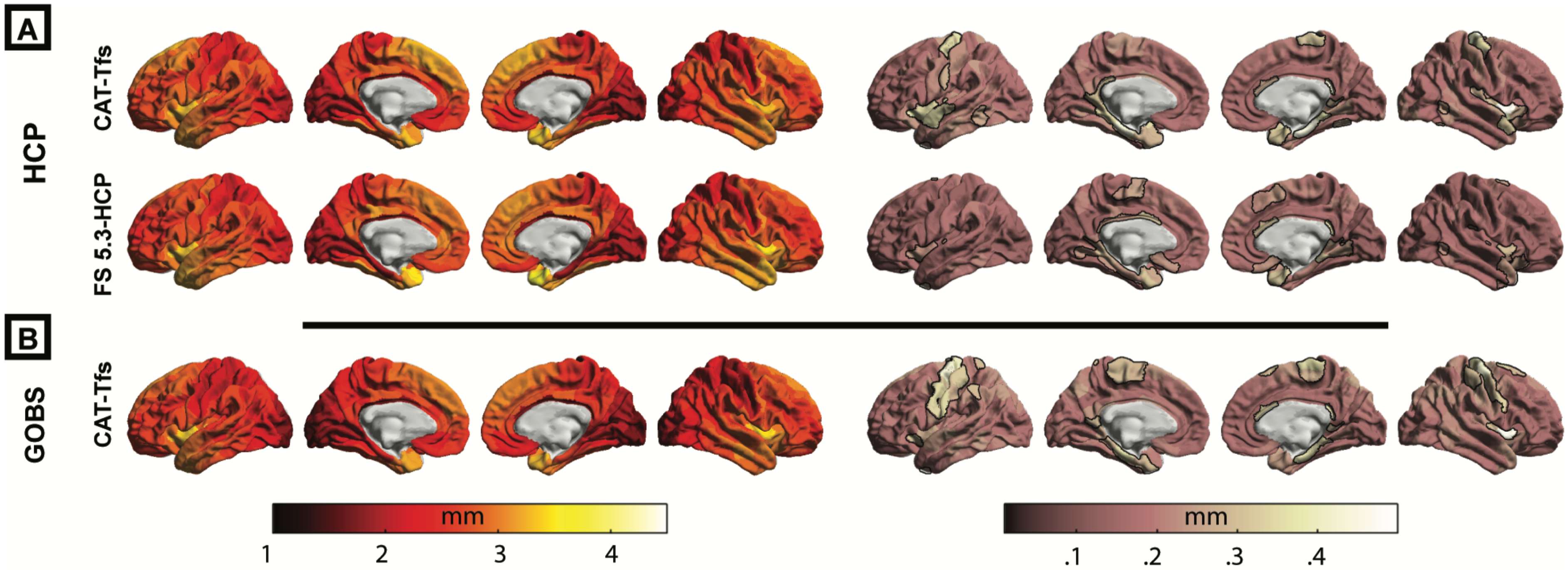

Supplementary Figure IV Regional mean (left columns) and SD (right columns) of cortical thickness estimates of CAT-Tfs and FreeSurfer v5.3-HCP (only for HCP sample) pipelines. For each pipeline, parcels with the highest SD (top 10%) are depicted with a black surrounding. CAT-Tfs: modified CAT thickness estimates-Tfs; FS: FreeSurfer.

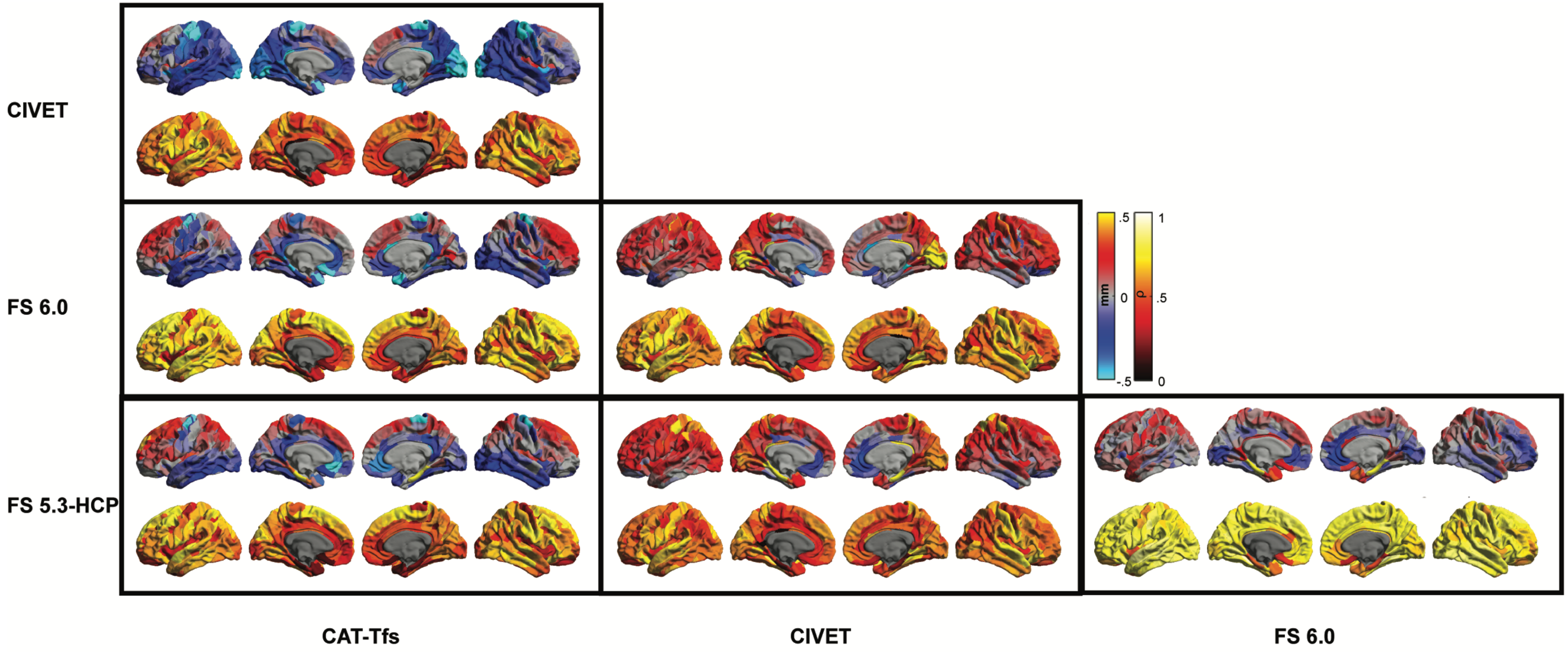

Supplementary Figure V Regional between-pipeline comparison of cortical thickness estimates within the HCP sample (modified CAT thickness estimates-Tfs). Top rows: Top rows: average paired-difference map of cortical thickness estimates; Red-yellow colors depict higher thickness estimate of the pipeline mentioned in column vs row and the dark-light blue depicts the opposite direction. Here only regions with significant paired t-test are shown ($p < 2 \times 10^{-5}$); Lower rows: Between-pipeline regional Spearman's correlation). CAT-Tfs: modified CAT thickness estimates-Tfs; FS: FreeSurfer.

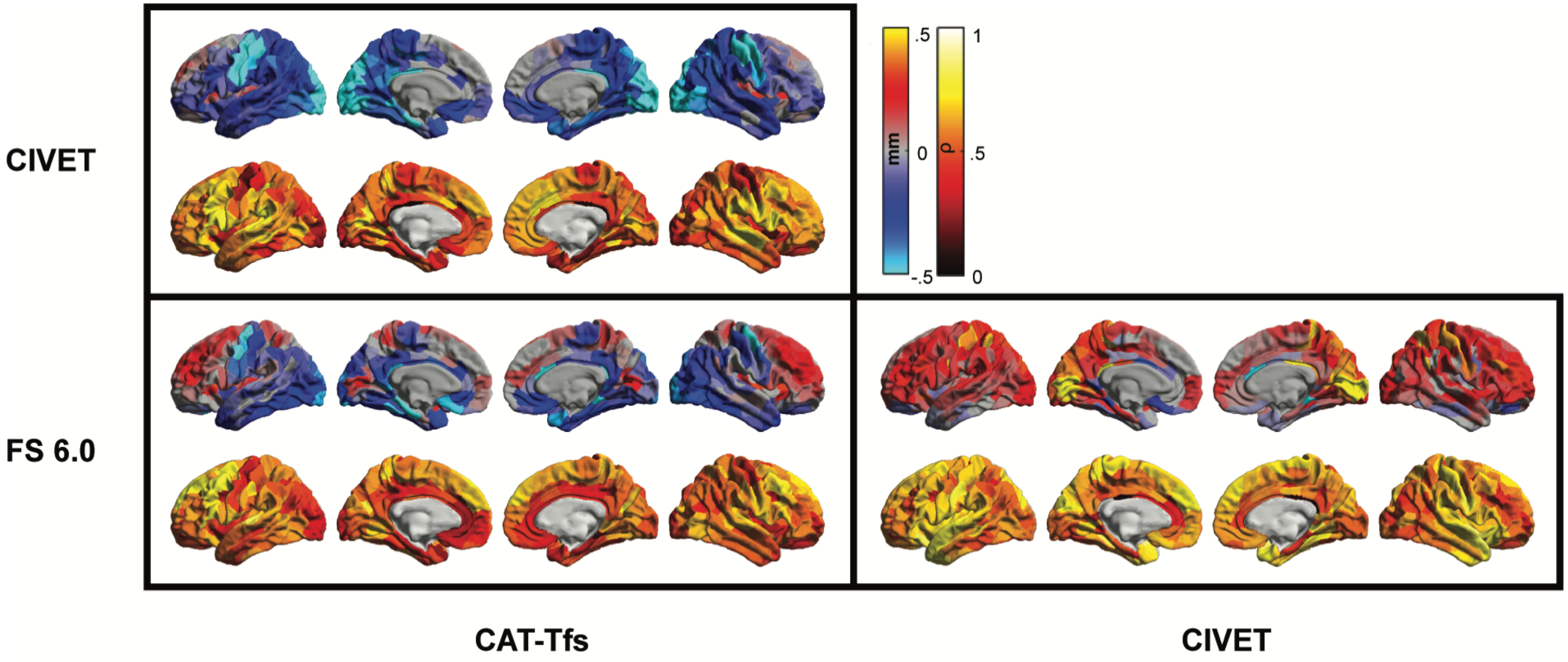

Supplementary Figure VI Regional between-pipeline comparison of cortical thickness estimates within the GOBS sample (modified CAT thickness estimates-Tfs). Top rows: average paired-difference map of cortical thickness estimates; Red-yellow colors depict higher thickness estimate of the pipeline mentioned in column vs row and the dark-light blue depicts the opposite direction. Here only regions with significant paired t-test are shown ($p < 4 \times 10^{-5}$); Lower rows: Between-pipeline regional Spearman's correlation). CAT-Tfs: modified CAT thickness estimates-Tfs; FS: FreeSurfer.

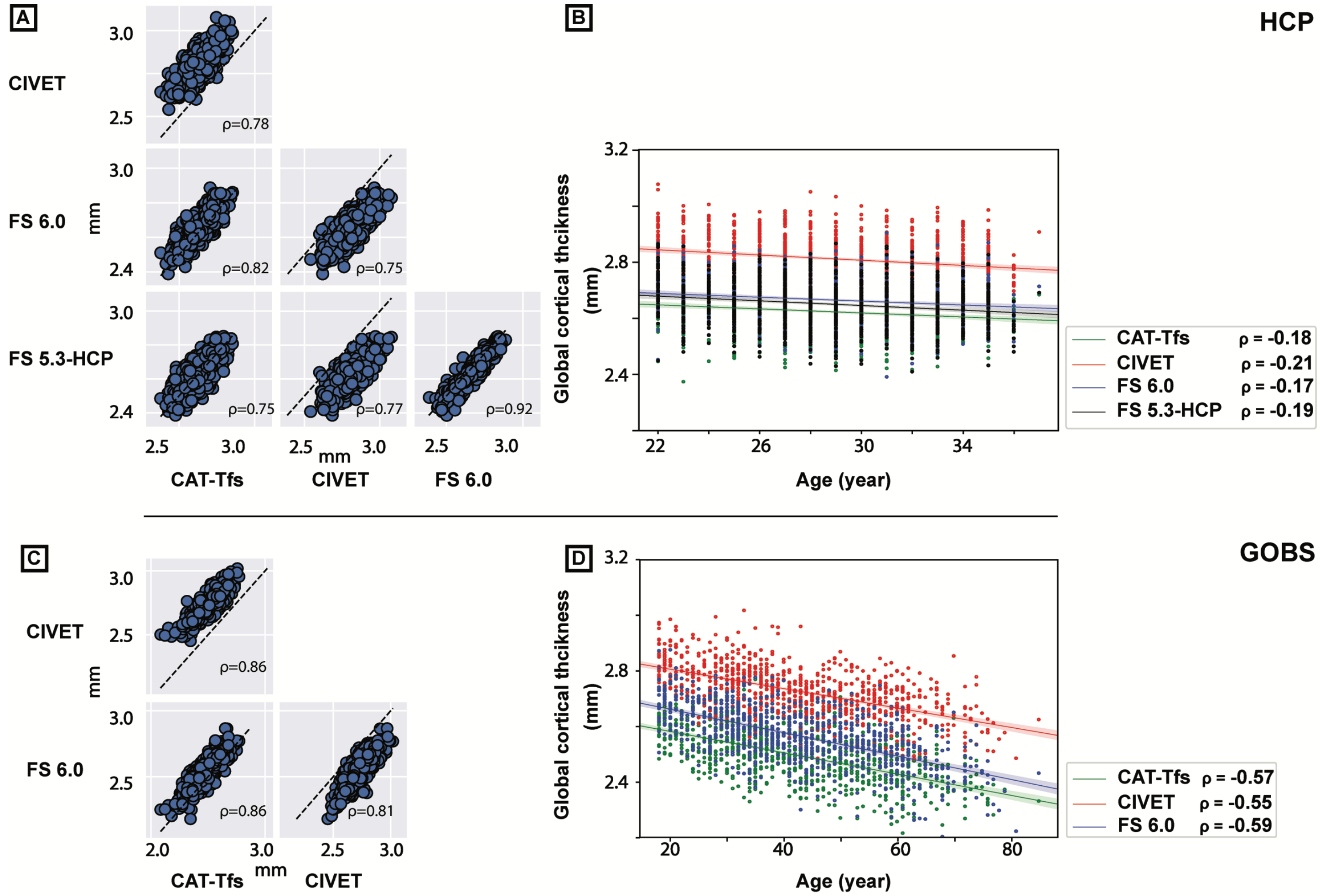

Supplementary Figure VII Between-pipeline comparison of global cortical thickness and its inter-individual variability. Scatter plots of pair-wise comparison of global cortical estimates and their Spearman's correlations, within two cohorts (A, C; dashed line depicts the identity line(y=x)). B, D: Scatter plots of association between participants' age and global cortical thickness from each pipeline, for each cohort. Legends depict Spearman's correlation between age and global thickness of each pipeline. CAT-Tfs: modified CAT thickness estimates-Tfs; FS: FreeSurfer.

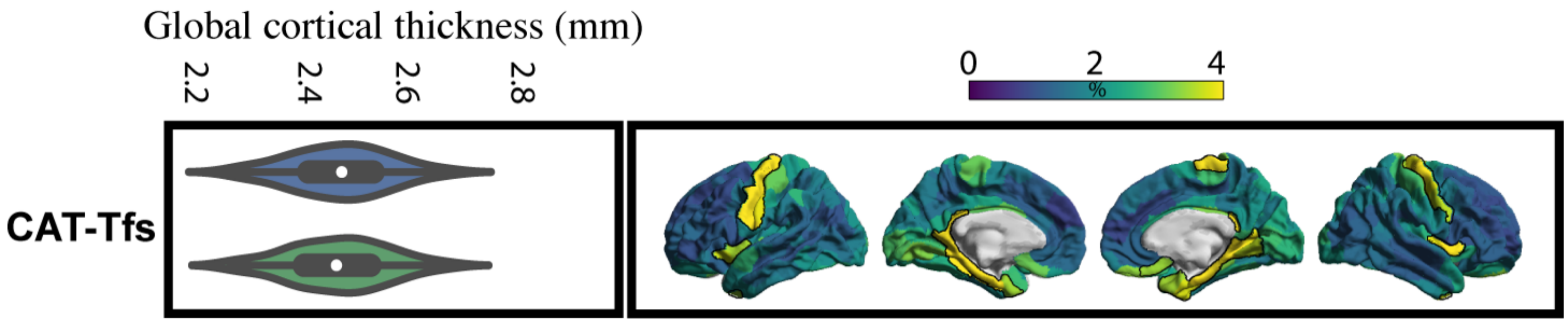

Supplementary Figure VIII Test-retest reliability. Left: pairs of violin plots demonstrate distribution of global cortical thickness estimates from odd and even scans, over all the subjects. Right: regional distribution of mean absolute percent error (MAPE) for each pipeline. Ten percent of the parcels, showing the lowest test-retest reliability, are surrounded with black line. The lighter colors in the spatial maps depict higher MAPE. CAT-Tfs: modified CAT thickness estimates-Tfs; FS: FreeSurfer.

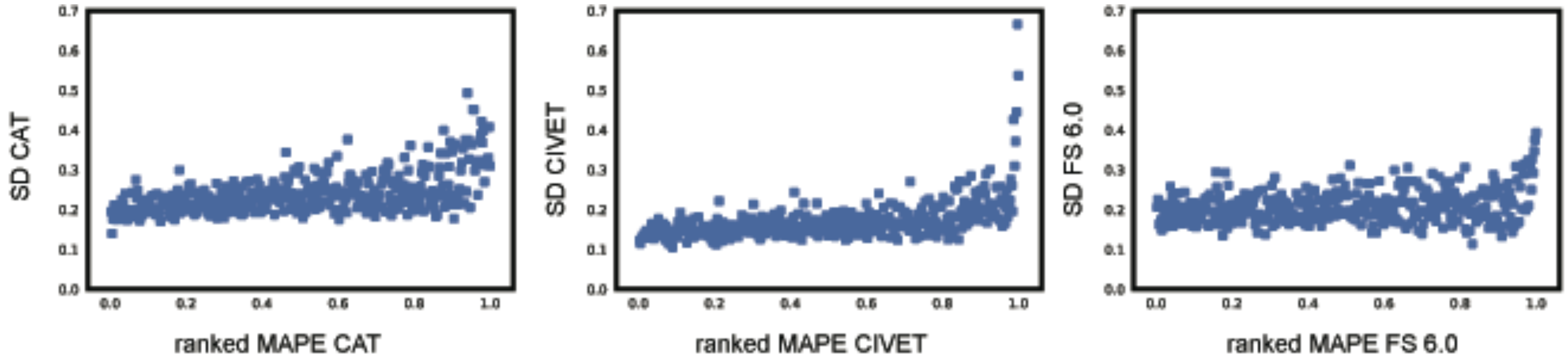

Supplementary Figure IX Relationship between standard deviation in each parcel and its rank in the MAPE distribution. In general, parcels with highest standard deviation (SD) across individuals, within the GOBS cohort, also demonstrated higher MAPE, i.e. more error in the test-retest experiment.

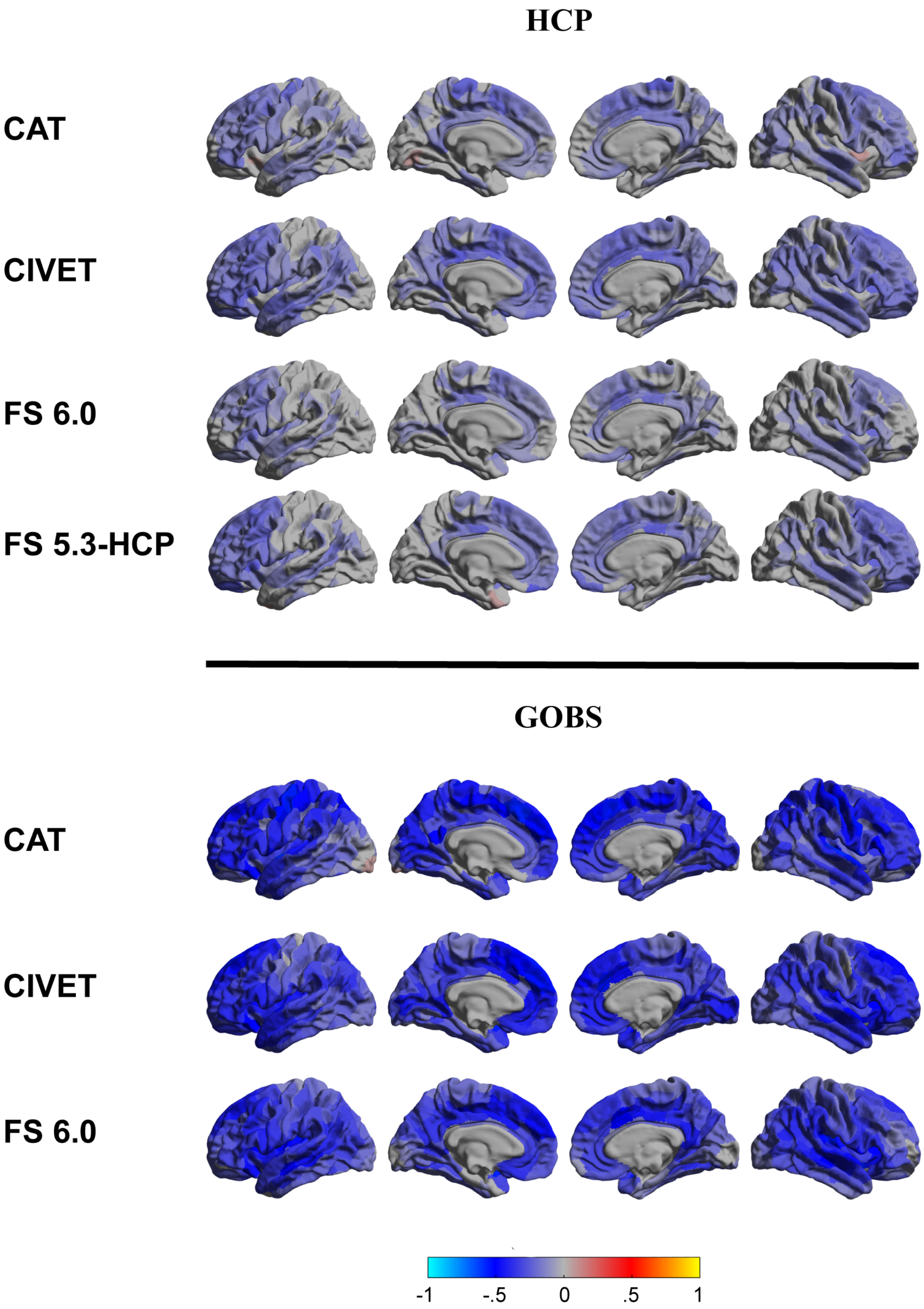

Supplementary Figure X Regional cortical thickness-age associations (color bar shows spearman's correlation).
